# Examining the Clinical Characteristics of Trigeminal Neuralgia at a Dental Hospital: A Prospective Study

**DOI:** 10.7759/cureus.34862

**Published:** 2023-02-11

**Authors:** Fatema Akhter

**Affiliations:** 1 Surgical and Diagnostic Sciences, Dar Al Uloom University, Riyadh, SAU

**Keywords:** dental patient, trigeminal neuralgia. age group, neuropathic pain, orofacial pain, lancinating pain, clinical profile

## Abstract

Background and objective

Trigeminal neuralgia (TN) is a paroxysmal shock-like neuropathic pain condition that causes sudden, typically unilateral, severe, brief, stabbing, and recurrent sensations in the distribution of one or more trigeminal nerve branches. Although the clinical profile of TN patients has been generally established, there has never been a study on the condition among the population of Northeast India. Furthermore, there is scarce research describing the clinical features of TN in patients who visit a dental college. In light of this, we conducted this study to assess the clinical characteristics and parameters of TN in an Indian population.

Methods

Medical records of 60 patients with TN were reviewed prospectively for patient demographics, characteristics of the pain, and treatment modalities.

Results

Of the 60 patients, 55% were male, and 45% were female. The peak incidence was found in the age range of 55-64 years. Pain occurred equally on the right and left sides of the face. The maxillary division of the trigeminal nerve was the most frequently affected (40%) region, followed by mandibular division (35%) and the combined maxillary and mandibular division alone (25%). Most patients described their attacks as shock-like (78.33%) and of spontaneous onset (86.67%).

Conclusion

In the present study, TN affected males more than females and occurred most frequently in patients aged 55-64 years. A comparative analysis of the pain characteristics between different age groups and genders is useful for the management of these patients.

## Introduction

Trigeminal neuralgia (TN) refers to a paroxysmal shock-like neuropathic pain condition that leads to sudden, typically unilateral, severe, brief, stabbing, and recurrent sensations in the distribution of one or more trigeminal nerve branches. It is frequently triggered by light stimuli in a trigger zone [1]. Classical TN is described by the International Headache Society (IHS) as "a unilateral condition marked by transient electric shock-like pains, abrupt in start and termination, limited to the distribution of one or more divisions of the trigeminal nerve" [2]. The so-called trigger points or zones, located ipsilateral to the painful area but may be in the same or a different division of the trigeminal nerve, are physically stimulated to start an attack [3,4]. Speaking, brushing one's teeth, washing one's face, and chewing are some of the triggers [1,4]. An attack could also be brought on by wind and cold water. According to a recent study, chatting (54%) and lightly caressing the face (79%) were the most common actions associated with the triggering of paroxysmal pain [5]. Most trigger zones were reported to be in the perioral and nasal regions [5]. The maxillary and/or mandibular divisions of the trigeminal nerve were the most frequently affected regions by discomfort [4,6-9]. The affected side's facial muscles typically contract (tic) involuntarily in response to pain attacks.

Because of this, TN has also been referred to as "tic douloureux" (painful contraction) [10]. It seriously impairs dental hygiene, daily routines, and quality of life, and patients frequently get depressed [11,12]. TN's etiology still remains a mystery. Theoretical explanations include traumatic trigeminal nerve compression by tumors or vascular abnormalities, herpes simplex infection, and demyelinating disorders [10]. One of the main contributing factors is thought to be a blood vessel's compression of the trigeminal nerve in the root entrance zone in the posterior fossa, which causes nerve damage [13-16]. The condition primarily affects hypertensive individuals and patients older than 50 years, and it is more prevalent in women [17]. The right side of the face is typically the sole side of the face affected by TN, and it occurs more frequently than in the left side in most patients [3,10]. The characteristics of the pain and reaction to carbamazepine are crucial in diagnosing the illness because the clinical examination of the patients may not reveal any obvious neurological impairments. Both therapeutic and surgical methods can be used to treat TN. If medications like carbamazepine, baclofen, and phenytoin are ineffective, surgical techniques are used [18]. Although the clinical profile of TN patients has been generally established, It has not been studied in a population of Northeast Indians. Moreover, there is scant research describing the clinical features of TN in patients visiting a dental college [19]. The aim of the current study was to compare the results in TN patients with those of other studies by analyzing the clinical characteristics of TN in a sample of Northeast Indian patients based on age distribution and gender.

## Materials and methods

After receiving approval from the institute's ethical committee (Lugansk State Medical University Committee, Ukraine, with LSMU study trial no UK23890531), a prospective study of patients arriving at the department of oral medicine and radiology was carried out from January 2022 to November 2022. Each patient provided written informed consent. Patients with a history and clinical presentation that met the IHS criteria for TN were included in the study. All the patients were informed about the study, and those who expressed a lack of interest in participating were excluded. Finally, 60 TN patients who were willing to participate and had sufficient data for inclusion were chosen for the study via convenient sampling. The sample size for the current study was selected based on earlier research on a related subject [2]. The first-time TN was identified in the chosen patients. A test dosage of carbamazepine was administered as part of pharmacotherapy during any doubtful clinical presentation. The study included patients who responded to the test dose.

After providing their personal information (name, age, gender, and address), all chosen patients underwent interviews involving a structured questionnaire to obtain data about their TN histories. It involved the side of the face, the trigeminal division, and pain radiation within or outside the division of the trigeminal nerve. A visual analog scale was used to measure the degree of pain. The McGill Pain Questionnaire was used to evaluate the severity of pain [20]. The pain was classified into acute, spontaneous pain, and discomfort related to dental work or illnesses. Assessment of the periodicity of pain included determining the frequency and length of each episode, and the length of the refractory phase, as well as the inciting, alleviating, and related factors. Qualified oral medicine specialists conducted a standard head and neck examination to determine the trigger points and performed sensory tests in the trigeminal nerve distribution. To rule out any probable dental and bone disorders, necessary radiographic studies were done.

Various clinical parameters were evaluated in male and female patients of various age groups (35-44, 45-54, 55-64, 65-74, and ≥75 years) independently based on the following aspects: side of the face affected, division of nerve, radiation of pain, the character of pain, and beginning of pain. The collected data were tabulated, and statistical analysis was performed on them. The mean, standard deviation (SD), confidence intervals (CI), and proportions were the statistical variables that were applied. The data were compared using the Chi-square test, and the level of significance was set at 5%. To compare the means between the samples, the t-test was utilized.

## Results

A total of 60 patients, 33 men (55%) and 27 women (45%), were included in the study. Males ranged in age from 35 to 84 years old, with a mean of 58.97 years, and females ranged in age from 37 to 85 years old, with a mean of 59.96 years (Table 1).

**Table 1:** Age and sex of patients

Variables	Male	Female	Total
Number of patients	33	27	60
Mean age (years)	58.97	59.96	59.41

Both the right and left sides were equally involved, i.e., the right side of the face was involved in 30 patients, and the left side of the face was affected in 30 patients. The left side was more commonly involved in females (16 patients), while the right was more commonly involved in males (19 patients). However, the difference was not statistically significant (p=0.194) (Table 2).

**Table 2:** Correlation between clinical characteristics and gender of the patients *Level of significance: p>0.05: not significant; p<0.05: significant; p<0.01: highly significant; p<0.001: very highly significant

Clinical characteristics	Gender, n (%)		Total, n (%)	P-value
	Female (n=27)	Male (n=33)		
The side of the face Involved				
Left	16 (59.26)	14 (42.24)	30 (50)	0.194
Right	11 (40.74)	19 (57.57)	30 (50)	
Total	(100)	(100)	60	
Division of nerve involved				
V2	8 (29.63)	16 (48.48)	24 (40)	0.26
V3	10 (37.04)	11 (33.33)	21 (35)	
V2-V3	9 (33.33)	6 (18.18)	15 (25)	
Total	(100)	(100)	60	
Radiation of pain				
Within the division	17 (62.96)	13 (39.39)	30 (50)	0.069
Outside the division	10 (37.04)	20 (60.61)	30 (50)	
Total	(100)	(100)	60	
Characteristics of pain (McGill’s Pain Questionnaire)				
Burning/lancinating	10 (37.04)	1 (03.03)	11 (18.33)	0.002*
Shock-like	17 (62.96)	30 (90.91)	47 (78.33)	
Throbbing	0	2 (06.06)	2 (03.33)	
Total	(100)	(100)	60	
Onset of pain				
Acute, spontaneous	23 (85.19)	29 (87.88)	52 (86.67)	0.76
Correlated with dental treatment	4 (14.81)	4 (12.12)	8 (13.33)	
Total	(100)	(100)	60	

According to age distribution, it was found that the left side was more affected in patients in the age groups of 55-64 and 65-74 years. On the other hand, the right side was more commonly affected in those in the age groups of 35-44, 65-74, and ≥75 years. The results were statistically significant (p=0.049) (Table 3).

**Table 3:** Correlation between clinical characteristics and age of the patients *Level of significance: p>0.05: not significant; p<0.05: significant; p<0.01: highly significant; p<0.001: very highly significant

Clinical characteristics	Age groups, n															Total	P-value
	35-44 years				45-54 years				55-64 years		65-74 years		≥75 years					
	The side of the face involved																	
Left	1					5			12		7		5			30	0.049*
Right	9					4			5		6		6			30	
Division of nerve involved																	
V2	2			2				9			6	4				24	0.238
V3	3			4				8			3	3				21	
V2-V3	4			3				1			4	3				15	
Radiation of pain																	
Within the division	4		5				8				7	6				30	0.947
Outside the division	6		4				9				6	5				30	
Characteristics of pain (McGill’s Pain Questionnaire)																	
Burning/lancinating	0		3							3	1			4		11	0.140
Shock-like	10		6							12	12			7		47	
Throbbing	0		0							0	0			2		2	
Onset of pain																	
Acute, spontaneous	7	9								16	10				10	52	0.211
Correlated with dental treatment	3	0								1	3				1	8	

**Figure 1:**
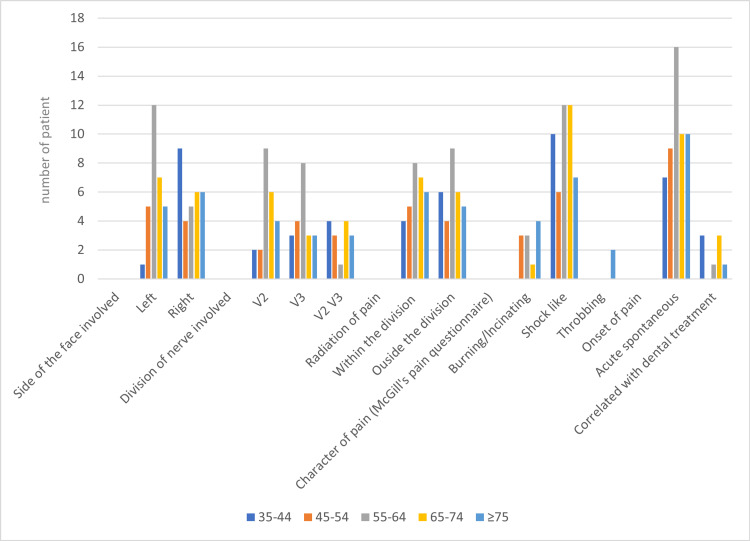
Correlation of clinical parameters at different age groups

The maxillary (V2) and mandibular (V3) divisions of the trigeminal nerve (containing both the right and left sides) were found to be implicated in 24 (40%) and 21 (35%) patients, respectively, while both V2 and V3 nerves were affected in the remaining 15 (25%) individuals (Table 2). Male patients (n=16) were more frequently affected by V2 division, whereas male and female patients were virtually equally affected by V3. Females had a higher prevalence of both V2 and V3 nerve involvement (n=9). However, the difference was not statistically significant (p=0.260) (Table 2). According to age distribution, it was found that both V2 and V3 nerves were commonly involved in patients in the age groups of 55-64 and 65-74 years. However, the results were not found to be statistically significant (p=0.238) (Table 3, Figure 1).

According to the assessment of pain radiation, the pain radiation occurred inside the division for 30 cases (50%), while it occurred outside the division for the remaining 30 cases (50%) (Table 2). It was observed that while there were more female patients with pain radiation inside the division (n=17), there were more male patients with pain radiation outside the division (n=20). However, the results were not statistically significant (p=0.069) (Table 2). The assessment of pain radiation in various age groups revealed that the majority of patients, with pain radiation both inside and outside the division, were between the ages of 55 and 64 and 65 and 74 years, with no statistically significant difference (p=0.947) (Table 3). The majority (n=47) of the patients (78.33%) who completed the McGill Pain Questionnaire reported feeling shock-like pain, while 11 patients (18.33%) reported experiencing searing, lancinating pain. Only two individuals (3.33%) reported experiencing throbbing pain. Most of the female patients (n=17) and nearly all the male patients (n=30) had shock-like pain, and there was a very significant difference between the groups (p=0.002) (Table 2).

Based on the age distribution among patients, it was discovered that shock-like discomfort affected patients of all ages, with patients in the age groups of 55-64 and 65-74 years reporting it the most frequently. However, the results were not statistically significant (p=0.140) (Table 3). The two categories - (1) acute, spontaneous onset and (2) association with dental therapy or disease - were used to analyze the pain's onset. The majority (n=52) of patients (86.67%) were found to have acute, spontaneous pain, whereas only eight patients (13.33%) had pain that started as a result of dental work or an illness. There was no statistically significant difference between men and women in this regard (p=0.760) (Table 2). Patients of all ages tended to experience acute, spontaneous pain, but the majority of them fell into the age groups of 55-64, 65-74, and 75+ years, but the findings were not statistically significant (p=0.211) (Table 3).

## Discussion

TN is a painful, uncommon condition that requires uninterrupted therapy and frequent neurosurgical intervention [21]. The first comprehensive account of TN was published in 1773 by John Fothergill. He described in detail the typical symptoms of the disease, including anxiety-related paroxysms of unilateral facial pain triggered by eating, speaking, or touching [22]. TN is still a clinical diagnosis based on a history of sharp, sudden pain that manifests as paroxysms or as isolated sensations and is separated by pain-free periods. The patient should ideally volunteer to give this description. However, many people who experience facial pain find it extremely difficult to explain their sensations with exact words. In this situation, the investigator may make as few prompts as possible and offer descriptive adjectives [12]. In this regard, it has been demonstrated that McGill's Pain Questionnaire is helpful in differentiating TN patients from those with other face problems [20].

In this study, 60 TN patients from a Northeastern Indian community were evaluated after having their general clinical characteristics and parameters assessed; 33 (55%) were men and 27 (45%) were women. However, other studies have reported TN was more frequent in females [3-7,17,21,23,24]. In the current study, the average age of all patients was 59.41 years, with 58.97 years for men and 59.96 years for women. This is consistent with the findings of previous studies that TN is more prevalent in older people with a mean age of 62.5 years and it primarily affects patients between the ages of 50 and 70 years [4,7,17,21,24,25]. Regarding the side of the face affected, it has been found that the right side was more frequently damaged than the left side in most studies [6,7,21,24,26]. They did not, however, assess the damaged side of the face separately for the different age groups and genders. The right and left sides were equally involved in the current investigation, meaning that while 50% (n=30) had the right side affected, the other 50% (n=30) had the left side affected. Left-side involvement was more common in female patients (59.26%), but right-side involvement was more common in male patients (57.57%). The difference, however, was not statistically significant (p=0.194).

The left side was more severely afflicted among patients in the age groups of 55-64 and 65-74 years with 40% (n=12) of those individuals being affected. However, patients in the age groups of 35-44, 65-74, and 75 years were more frequently affected on the right side, with 30% (n=9) of people in the 35-44 age group alone (p=0.049) being affected. The results were determined to be statistically significant. Although the numbers were lower than for older patients, the study demonstrates that patients in the younger age groups are also significantly affected by TN. This may be attributed to the fact that more hypertensive individuals are diagnosed in younger age groups today. It was discovered that the maxillary (V2) and mandibular (V3) divisions of the trigeminal nerve (containing both the right and left sides) were involved in 24 (40%) and 21 (35%) patients, respectively, while both V2 and V3 nerves were affected in the remaining 15 (25%) patients. These findings are in line with the studies by Katusic et al., Barker et al., and Dia Tine et al., which claimed that the maxillary division was the most affected branch (V2) [6,8,9]. On the other hand, the mandibular division (V3) of the trigeminal nerve was discovered to be the most commonly affected branch by Loh et al. [7] and Jainkittivong et al. [24]. The same discovery was made by all investigators; nevertheless, combinations of two or three divisions were less prevalent [27].

When analyzing the specific nerve divisions involved based on gender and age range, V2 involvement was found in 16 male patients at a rate of 48.48%, while V3 was in affected male and female patients at a rate of 33.33% and 37.04%, respectively. Involvement of both V2 and V3 nerves occurred more frequently in females (33.33% (n=9) than in males (18.18%) (n=6). The distinction was not, however, statistically significant (p=0.260). Furthermore, it was discovered that patients in the 65-74 and 55-64 age groups more frequently had V2 and V3 nerve involvement respectively. However, it was determined that the results were not statistically significant (p=0.238). These findings are in line with previous studies that also demonstrated the involvement of V2 and V3 nerve associated with different age groups as in our study [4,7,8]. The incidence of V2 and V3 involvement might be due to the somatotopic distribution of the sensory fibers of the trigeminal nerve.

The pain radiation occurred inside the division for 30 cases (50%), while it occurred outside the division for the remaining 30 cases (50%). Female patients (n=17) were found to have it more frequently within the division (62.96%), whereas male patients (n=20) had it more frequently outside the division (60.61%). However, the results were not statistically significant (p=0.069). According to the assessment of pain radiation in various age groups, the majority of patients with pain radiation inside and outside the division were between the ages of 55 and 64 and 65 and 74 years, respectively, with no statistically significant difference (p=0.947). Previous studies have reported that while radiation of pain within and outside the division occurred, that within the division was more frequently observed [6-8,28].

In the present study, it has been demonstrated that McGill's Pain Questionnaire is helpful in differentiating TN patients from those with other face problems [20]. According to the McGill Pain Questionnaire, the majority of the patients in the current study (n=47, 78.33%) had shock-like pain, whereas just 11 patients (18.33%) had searing, lancinating pain. Only two individuals (3.33%) reported experiencing throbbing pain. Most of the female patients (n=17, 62.96%) and nearly all the men (n=30, 90.91%) felt discomfort similar to shock. A highly significant difference between the groups was evident in the data (p=0.002).

Based on the distribution of patients' ages, it was discovered that shock-like discomfort affected patients of all ages, with patients in the 55-64 and 65-74 age groups reporting it the most frequently. However, the results were not statistically significant (p=0.140).

However, the Jainkittivong et al. study indicated that the majority of patients (77.6%) described their attack as a sharp pain [24]. In addition to electric shock-like discomfort, other descriptions of pain included stabbing pain (9.6%), numbness (6.9%), throbbing pain (6.4%), and scorching pain (4.2%) [22]. Only three types of pain - burning/lancinating, shock-like, and throbbing - were available for the patients to choose from in the current investigation, which used McGill's Pain Questionnaire. There was no classification for acute pain. In contrast, patients in the study by Jainkittivong et al. freely described their level of pain. As a result, there might be variations in the language used to describe the pain. Sometimes, TN pain is incorrectly attributed to a dental cause [29]. Therefore, extreme caution should be exercised as TN may initially be mistaken for an episodic toothache. Dentists can quickly rule out the pain of odontogenic origin because odontalgia can be treated with a local anesthetic [24].

In the current investigation, two categories - that of acute, spontaneous onset and that associated with dental therapy or disease - were used to evaluate the beginning of pain. The majority (n=52) of patients (86.67%) reported acute, spontaneous pain, whereas only eight patients (13.33%) reported pain that started as a result of dental work or an illness. There was no statistically significant difference in the prevalence of acute spontaneous onset between males (87.88%) and females (85.19%) (p=0.760). Patients of all ages tended to experience acute, spontaneous pain, but the majority of them fell into the age groups of 55-64, 65-74, and 75+ years, but the findings were not statistically significant (p=0.211). The findings are in line with those of the studies by Zakrzewska et al. [1] and Loeser [4]. The majority of the study participants claimed that their pain was not brought on by dental work or illness but rather by some physical stimulus. As a result, patients could avoid the stimuli and skip regular oral hygiene appointments. In addition, it was noted by Türp and Gobetti [30] and Jainkittivong et al. [24] that many TN patients had lost teeth due to unneeded extractions. Many such symptomatic teeth have been lost due to this frequent false diagnosis, which has misled both patients and dentists into believing that the pain was odontogenic in origin. Despite the dentists' warnings that pulling the painful teeth might not always help, a number of individuals insisted on having their teeth removed [24].

This study has a few limitations, primarily its small sample size. Further multicentric studies with larger sample sizes are required to better understand the nature of this crippling illness.

## Conclusions

In this study, we analyzed the general clinical features and characteristics of 60 TN patients from a Northeast Indian community. Males were more commonly impacted by TN than females in the current study, and patients between the ages of 55 and 64 years were the most frequently affected group. Most TN patients were initially treated by dentists because the majority of pain complaints started in the oral cavity. Dentists should be educated about the nature and clinical features of TN to prevent mistreatment. The patient should be promptly sent to orofacial or oral pain medicine specialists in doubtful situations.
